# Ownership and usage of mosquito nets after four years of large-scale free distribution in Papua New Guinea

**DOI:** 10.1186/1475-2875-11-192

**Published:** 2012-06-10

**Authors:** Manuel W Hetzel, Gibson Gideon, Namarola Lote, Leo Makita, Peter M Siba, Ivo Mueller

**Affiliations:** 1 Papua New Guinea Institute of Medical Research, PO Box 60, Goroka, EHP, 441, Papua New Guinea; 2 The University of Queensland, School of Population Health, Herston, Qld, 4006, Australia; 3 National Department of Health, Port Moresby, Papua New Guinea; 4 Walter and Eliza Hall Institute of Medical Research, Melbourne, Australia; 5 Barcelona Centre for International Health Research (CRESIB, Hospital Clínic-Universitat de Barcelona), Barcelona, Spain

**Keywords:** Malaria, Insecticide-treated nets, Papua New Guinea

## Abstract

**Background:**

Papua New Guinea (PNG) is a highly malaria endemic country in the South-West Pacific with a population of approximately 6.6 million (2009). In 2004, the country intensified its malaria control activities with support from the Global Fund. With the aim of achieving 80% ownership and usage, a country-wide campaign distributed two million free long-lasting insecticide-treated nets (LLINs).

**Methods:**

In order to evaluate outcomes of the campaign against programme targets, a country-wide household survey based on stratified multi-stage random sampling was carried out in 17 of the 20 provinces after the campaign in 2008/09. In addition, a before-after assessment was carried out in six purposively selected sentinel sites. A structured questionnaire was administered to the heads of sampled households to elicit net ownership and usage information.

**Results:**

After the campaign, 64.6% of households owned a LLIN, 80.1% any type of mosquito net. Overall usage by household members amounted to 32.5% for LLINs and 44.3% for nets in general. Amongst children under five years, 39.5% used a LLIN and 51.8% any type of net, whereas 41.3% of pregnant women used a LLIN and 56.1% any net. Accessibility of villages was the key determinant of net ownership, while usage was mainly determined by ownership. Most (99.5%) of the household members who did not sleep under a net did not have access to a (unused) net in their household. In the sentinel sites, LLIN ownership increased from 9.4% to 88.7%, ownership of any net from 52.7% to 94.1%. Usage of LLINs increased from 5.5% to 55.1%, usage of any net from 37.3% to 66.7%. Among children under five years, usage of LLINs and of nets in general increased from 8.2% to 67.0% and from 44.6% to 76.1%, respectively (all p ≤ 0.001).

**Conclusions:**

While a single round of free distribution of LLINs significantly increased net ownership, an insufficient number of nets coupled with a heterogeneous distribution led to overall low usage rates. Programme targets were missed mainly as a result of the distribution mechanism itself and operational constraints in this very challenging setting.

## Background

Malaria transmission is endemic in all lowland areas of Papua New Guinea (PNG) and high morbidity caused by both falciparum and vivax malaria presents a major burden to the population and the local health services [1]. Attempts were made in the 1960s and 1970s to eliminate malaria from PNG territory with a mix of indoor residual spraying (IRS) with dichlorodiphenyltrichloroethane (DDT), mass drug administration and environmental measures [2,3]. Coverage of 53% of the population was achieved in 1973 leading to near elimination in many highland areas and significant reductions in coastal areas covered by the programme [2,4,5]. However, due to operational constraints such as extremely difficult accessibility of large parts of the country, diminishing support of communities [6], increasing resistance to commonly used drugs [7,8] and changes in mosquito biting behaviour [9], the gains proved unsustainable. The elimination target was subsequently abandoned with the 1974–78 National Health Plan [10] and in the 1980s, large-scale IRS operations ceased [11].

In 1985, the Papua New Guinea Institute of Medical Research (PNG IMR) demonstrated in one of the first trials worldwide the health impact of treating mosquito nets with insecticide [12] and, later, a protective mass effect of mosquito nets in a highly endemic area in East Sepik province [13]. The need for personal protection in mosquito infested areas of PNG had been recognized long before, be it by troops during the Pacific War 1940–45 [2] or by certain local communities that used “mosquito baskets” to protect themselves from mosquito bites at night [14]. The National Malaria Control Programme started recommending the use of insecticide-treated nets (ITN) in 1989, but net distributions remained few and far between and no quick scaling-up of ITNs was envisaged [15].

In 2004, the PNG National Department of Health (NDoH) managed to secure a first malaria grant from the Global Fund to Fight AIDS, Tuberculosis and Malaria (GFATM) allowing it to re-intensify its malaria control efforts. The emphasis of the grant was on nationwide availability of free long-lasting insecticide-treated nets (LLIN) as main preventive measure, in line with the new National Health Plan 2001–10 [16]. The grant supported an entirely campaign-based strategy that aimed to quickly achieve high levels of ownership and usage of nets (“catch-up” strategy). The entire country should be covered with a single round of free LLIN distribution between 2004 and 2009. The number of LLINs required was estimated based on the year 2000 national census (with a projected 2.7% annual population growth) and the aim was to achieve 80% household ownership and 80% usage in children under the age of five years and pregnant women with a distribution rate of one LLIN per 2.5 people [17].

The distribution campaign was implemented jointly by the non-governmental organization Rotarians Against Malaria (RAM), the NDoH and provincial and district health authorities. RAM imported and delivered the LLINs to provincial or district headquarters where the distribution to the household level was taken over by the local health authorities. Over the five-year grant period, a total of 2,321,100 LLINs (PermaNet®, Vestergaard Frandsen) were supplied by RAM (Anna Maalsen, NDoH, personal communication). By the end of the grant (31 July 2009), 2,005,052 LLINs had been distributed to the household-level under the auspices of provincial and district health authorities (Anna Maalsen, NDoH, personal communication; 1.352 million reported in the GFATM grant performance report [18]). In two provinces (Enga and Southern Highlands), the household-level distribution had not been completed due to operational problems. Over the entire grant period, one LLIN had been distributed per 3.31 people (2,005,052 LLINs for 6,641,268 projected 2009 population, based on Census 2000 data + province-level annual population growth of 1.8-3.5% [19]). The distribution was not accompanied by a major behaviour change campaign. Malaria awareness creation beyond rather informal interpersonal communication during the net distribution did not markedly increase over the course of the grant.

The aim of this study was to assess the outcome of the nationwide campaign-based delivery and evaluate ownership and usage indicators against pre-distribution values and stated programme targets.

## Methods

### Study design and sites

Between October 2008 and August 2009, a country-wide cross-sectional household survey was carried out, collecting household and individual level data on ownership and usage of mosquito nets. Selection of study households was based on a stratified multi-stage random sampling procedure. Administrative organizational units (province, district, and village) were used as sampling stages.

To assess the national-level campaign outcome, a sample was drawn from the 17 (of 20) provinces that had already been covered with the campaign. In fifteen provinces, two districts were randomly selected; in two provinces, only one district was eligible for selection. In each district, two villages were randomly sampled from a geo-referenced village database. If a selected village was very difficult to reach (i.e. accessible only by helicopter or more than one day walk), it was replaced by another randomly sampled village.

In addition to the country-wide survey, a before-after assessment of the distribution was conducted in six sentinel sites. Sentinel site locations were purposively selected based on operational and epidemiological considerations from provinces and districts that had not yet been covered with the distribution campaign in 2008. In each sentinel site, household surveys were carried out in three or four randomly selected villages located within the catchment area of a sentinel health centre. In this paper, the sentinel site names refer to the location of the health centre. A baseline survey was conducted prior to the LLIN distribution in 2008/09 and a follow-up survey in the same villages one year after the baseline.

In each study village, 30 to 35 households were randomly sampled upon arrival of the survey team. The sampling frames were household lists established jointly by village leaders and the survey team. All individuals in a selected household were included into the survey. The procedures for sampling households and data collection were identical for country-wide and before-after surveys.

### Data collection

A structured questionnaire was administered to the heads of sampled households by two teams of trained field interviewers. The questionnaire largely followed the design of the Malaria Indicator Survey household questionnaire [20]. Demographic characteristics of all household members were recorded alongside accounts of mosquito net ownership and usage. Where possible, ownership was verified by the field interviewers who checked the presence and the type of nets. Net usage referred to the night prior to the interview. Participation in the survey was voluntary and interviews conducted only upon verbal consent. The village locations were recorded using handheld GPS devices (Garmin etrex, Garmin Ltd., Olathe, Kansas, USA).

### Data analysis

All data collection forms were double-entered into a Microsoft FoxPro (Microsoft Corporation) database at the PNG IMR in Goroka and analysed with Intercooled Stata 10.1 (StataCorp LP, College Station, USA).

For household level indicators derived from country-wide surveys, unweighted and weighted proportions were estimated. Overall weights were calculated as an inverse of an observation’s probability of selection. To account for the staged sampling strategy, the overall probability of selection was calculated as a product of the selection probabilities at each sampling stage, i.e. the probability of a district being selected within a province, the probability of a village being selected within a district, and the probability of a household being selected within a village. Since all individuals of a sampled household were eligible, individual level weights equalled the weights of the household an individual belonged to.

The Stata survey design command set (“svy” prefix) was used to allow for complex survey design in univariate and multivariate analyses. The application of the survey command set applies survey weights to all calculations and adjusts standard errors to the multi-stage sampling strategy [21].

Linear regression was used to assess differences in means between strata. Logistic and linear regression models were used to determine factors determining net ownership and usage based on the country-wide post-distribution survey. Goodness-of-fit of logistic regression models was tested by using the “svylogitgof” command in Stata which takes into account weights and sampling strategy [22].

All national and regional estimates are based on weighted analyses, while the before-after assessment of data from purposively selected sentinel sites did not apply weights.

### Ethical considerations

The study protocol was approved by the Institutional Review Board of PNG IMR (IMR IRB No. 0803) and the PNG Medical Research Advisory Committee (MRAC No. 07.30, 30 November 2007).

## Results

### Study sample

**Figure 1:**
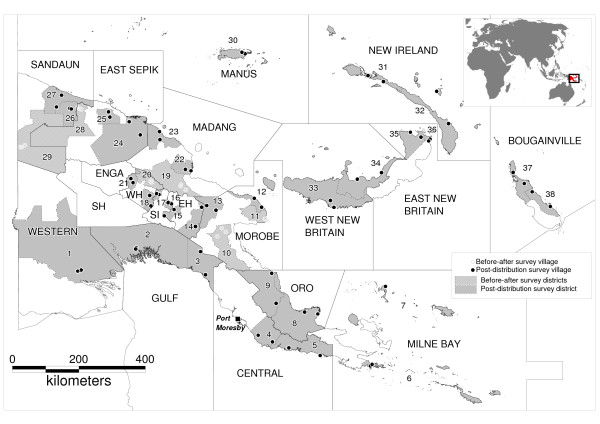
**Location of study sites.** Provinces are labelled in capitals; EH Eastern Highlands, SH Southern Highlands, SI Simbu (Chimbu), WH Western Highlands. Study districts: 1 Middle Fly, 2 Kikori, 3 Kerema, 4 Rigo, 5 Abau, 6 Samarai-Murua, 7 Kiriwina-Goodenough, 8 Ijivitari, 9 Sohe, 10 Bulolo, 11 Finschhafen, 12 Tewai-Siassi, 13 Markham, 14 Obura-Wonenara, 15 Lufa, 16 Chuave, 17 Kerowagi, 18 Angalimp-South Waghi, 19 Usino-Bundi, 20 Jimi, 21 Baiyer-Mul, 22 Madang, 23 Bogia, 24 Angoram, 25 Yangoru-Saussia, 26 Nuku, 27 Aitape-Lumi, 28 Ambunti-Dreikikir, 29 Telefomin, 30 Lorengau, 31 Kavieng, 32 Namatanai, 33 Kandrian-Gloucester, 34 Talasea, 35 Gazelle, 36 Kokopo, 37 North Bougainville, 38 Central Bougainville.

The country-wide household survey included 64 villages in 32 districts in 17 provinces (Figure 1). Based on regional climatological profiles, the surveys coincided with the rainy season in 35 villages, the end of the rainy and beginning of the dry season in 18 villages and the middle of the dry season in 11 villages [23]. However, weather patterns vary greatly within each region and were not necessarily consistent with the predicted season. Forty-eight (75%) of these villages were accessible by vehicle (on a road or bush track) from the centre to which bulk nets had been delivered by RAM. Out of the remaining 16 villages, eight were accessible by boat, three by charter plane, and five by charter plane plus boat. A total of five villages could only be reached on foot.

Fifty (78.1%) villages were located below 1,300 m altitude where climatic conditions are generally favourable for endemic malaria transmission. Another 9 (14.1%) were located at altitudes of unstable transmission between 1,300 m and 1,700 m, where malaria epidemics may occur, and five (7.8%) villages were above 1,700 m where malaria transmission is unlikely [1].

A total of 1,958 households were included in the study and the household heads were interviewed (median per village: 30; interquartile range [IQR] 30, 32.25). Individual level usage data for the night before the survey was collected for 10,258 household members (median per village: 155.5; IQR 133.75, 187.5), including 1599 children under five years of age (15.6% of all individuals with reported age) and 132 pregnant women aged 15 to 49 years (5.3% of all women in this age group). The average household size was 6.6 residents.

Household surveys for before-after assessment were conducted in six sentinel surveillance sites (in five different provinces) and included 19 villages (Figure 1). A total of 15 (78.9%) of these villages were located below 1,300 m altitude and only four (21.1%) between 1,300 and 1,700 m. In the pre-distribution survey in 2008/09, 596 households were visited (median per village 30; IQR 30, 35), covering 2,825 household members (median per village 150; IQR 131.5, 166) including 390 (13.9%) children under five years of age and 53 pregnant women aged 15 to 49 years (7.7% of all women in this age group). The follow-up survey in the same villages covered an independent random sample of 645 households (median per village 37; IQR 29.5, 41) with 3,303 household members (median per village 180; IQR 151, 204.5) including 482 (14.7%) children under five years and 47 pregnant women (5.5%).

### Household ownership of mosquito nets

Countrywide, 64.6% (95% CI 55.5-72.7) of households owned at least one LLIN while 80.1% (75.1-84.3) owned any type of mosquito net. The average number of nets per household was 1.3 (1.1-1.5) for LLINs and 1.8 (1.6-1.9) for any type of net. This resulted in an average of 3.4 (3.1-3.7) people per LLIN or 3.0 (2.8-3.3) people per net of any type (Table 1).

**Table 1:** Key indicators of mosquito net ownership

**Background characteristics**	**Households with at least one net**	**Average number of nets per household**	**Average number of household members per net**	**Households with at least one LLIN**	**Average number of LLINs per household**	**Average number of household members per LLIN**	**Number of households**
	**% (95% CI)**	**Mean (95% CI)**	**Mean (95% CI)**	**% (95% CI)**	**Mean (95% CI)**	**Mean (95% CI)**	
region							
Southern	79.8 (63.0-90.2)	2.2 (1.9-2.6)	2.7 (2.3-3.0)	64.5 (42.4-81.7)	1.6 (1.1-2.2)	3.0 (2.8-3.2)	528
Highlands	70.7 (60.1-79.4)	1.3 (0.9-1.7)	3.4 (2.7-4.1)	56.3 (37.2-73.7)	1.0 (0.5-1.5)	3.6 (2.5-4.7)	386
Momase	95.0 (88.5-97.9)	2.0 (1.9-2.2)	2.8 (2.6-3.0)	68.7 (48.0-83.8)	1.2 (0.9-1.6)	3.5 (3.2-3.9)	472
Islands	78.1 (70.2-84.4)	1.7 (1.4-1.9)	3.2 (2.9-3.5)	70.2 (61.9-77.4)	1.4 (1.2-1.7)	3.4 (3.0-3.8)	572
P-value	0.003*	0.008^§^	0.084^§^	0.529*	0. 319^§^	0.044^§^	
Total	80.1 (75.1-84.3)	1.8 (1.6-1.9)	3.0 (2.8-3.3)	64.6 (55.5-72.7)	1.3 (1.1-1.5)	3.4 (3.1-3.7)	1958

Based on weighted analysis of country-wide post-distribution survey data.

Significant regional differences in household ownership of any net were observed with the highest ownership in Momase (95.0%) and the lowest in the Highlands region (70.7%). The overall statistical significance resulted from the differences between Momase and all other regions (vs. Southern p = 0.020; vs. Highlands p = 0.002; vs. Islands p = 0.004). While differences were significant for nets of any type, they were not for LLINs, suggesting variations in pre-campaign ownership but not necessarily in campaign coverage. The average number of nets per household also differed between regions reaching statistical significance for nets of any type (p = 0.008), but not for LLINs (p = 0.319). In Southern region, the number of nets of any type and number of LLINs per household was highest. Taking into account the household size, this led to the lowest person/net and person/LLIN ratios in Southern region. The regional differences in person/LLIN ratios were statistically significant (p = 0.044) as a result of the difference between Southern and Momase/Islands regions (Table 1). Considering that the largest average household size was measured in Southern region (six household members vs. five in Highlands, 5.5 in Momase, 5.7 in Islands), this region must have received more nets per household and per person as compared to all other regions.

**Figure 2:**
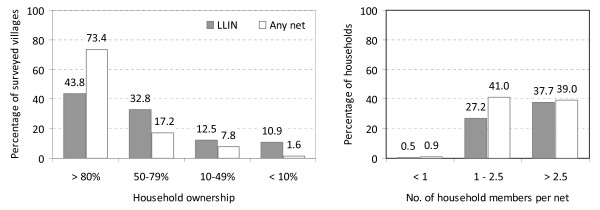
LLIN ownership at village (left) and household levels (right).

Ownership levels were very heterogeneous between and within villages. Out of the 64 villages surveyed after the distribution campaign, 28 (43.8%) had reached the household LLIN coverage target of 80% and above; 47 (73.4%) had 80% ownership of nets of any type. On the other hand, in three villages (4.7%) none of the households had a LLIN and seven villages (10.9%) had less than 10% LLIN coverage (the seven were located in seven different provinces). A total of 542 (27.7%) households were covered with a ratio of one LLIN per 2.5 or fewer household members (the original distribution ratio). In 739 (37.7%) households more than 2.5 people shared one LLIN and ten households had more LLINs than household members (Figure 2).

### Usage of mosquito nets

Overall usage by household members amounted to 32.5% (27.0-38.4) for LLINs and 44.3% (38.8-49.9) for any type of net. Usage was higher in the target group of children under five years of age, with 39.5% (32.8-46.5) having slept under a LLIN the previous night and 51.8% (45.4-58.1) under any type of net (Table 2). Usage of LLINs by pregnant women was 41.3% (31.6-51.8), whereas 56.1% (44.1-67.5) had slept under any type of net.

**Table 2:** Mosquito net usage by household members

**Background characteristics**	**Used any mosquito net the previous night % (95% CI)**	**Used LLIN the previous night % (95% CI)**	**Number of household members**^‡^
Age (years)			
<5	51.8 (45.4-58.1)	39.5 (32.8-46.5)	1599
5-14	43.2 (36.9-49.7)	30.9 (24.9-37.5)	2959
15+	42.8 (37.2-48.5)	31.3 (25.9-37.4)	5680
P-value^*^	0.003	0.004	
Male	42.3 (36.7-48.1)	31.5 (26.0-37.5)	5171
Female	46.3 (40.7-51.9)	33.5 (27.9-39.6)	5087
P-value^*^	0.001	0.048	
Southern	52.5 (40.7-64.0)	40.4 (29.9-51.9)	3041
Highlands	30.6 (22.8-39.8)	22.7 (14.0-34.6)	1702
Momase	74.0 (64.3-81.8)	47.0 (34.1-60.4)	2364
Islands	29.3 (18.7-42.6)	25.4 (16.5-36.8)	3151
P-value^*^	<0.001	0.007	
Total	44.3 (38.8-49.9)	32.5 (27.0-38.4)	10 258

Based on weighted analysis of country-wide post-distribution survey data.

* Chi-square test.

^‡^ sleeping in the house the night before the survey.

Net usage was significantly higher in children under five years of age than in older age groups both for nets of any type (51.8% vs. 42.9%, p < 0.001) and for LLINs (39.5% vs. 31.2%, p < 0.001). Female household members above 15 years of age more frequently slept under any net (46.1% vs. 39.3%, p < 0.001) or a LLIN (33.3% vs. 29.3%, p = 0.011) than their male counterparts; no difference was observed at younger age.

Significant regional differences were observed in overall usage of nets of any type and LLINs in particular (Table 2). Usage in the target groups of children under five and pregnant women followed the same trend and were statistically significant (all p < 0.05) except for use of LLINs by pregnant women (p = 0.067). Highest usage levels were achieved in Momase region (74.0% nets in general, 47% LLINs) and lowest in the Highlands (30.6% nets in general, 22.7% LLINs) and the Islands (29.3% nets in general, 25.4% LLINs). The significant regional differences in LLIN usage contrast with LLIN ownership which showed no significance in regional variation (Table 1). Interestingly, households in Momase and Islands regions had very similar LLIN ownership levels (68.7% and 70.2%) and person/LLIN ratios (3.5 and 3.4) but the usage differed significantly with Momase achieving 47% overall usage and Islands only 25.4%. In Islands region, the contrast between high ownership and low usage is equally striking for nets of any type (78.1% vs. 29.3%) (Tables 1 and 2).

### Determinants of mosquito net ownership and usage

Determinants of net ownership and usage were assessed based on the country-wide post-distribution survey. Multivariate logistic regression models found that the principal determinant of household net ownership was the accessibility of a village and the number of people in a particular household. Households in villages accessible only by air were significantly less likely to own a LLIN (odds ratio [OR] = 0.19) or a net of any type (OR = 0.26). Households with more than one household member were more than five times more likely to own any net (OR = 5.35) or a LLIN (OR = 5.26) than households with a single person. Regional differences in ownership were explained mainly be these two variables with the exception of ownership of any net, which was slightly more probable in Momase region even after adjusting for other factors (Table 3).

**Table 3:** Determinants of mosquito net and LLIN ownership

**Variable**	**Net ownership**				**LLIN ownership**			
	**Crude OR (95% CI)**	**p**	**Adj. OR (95% CI)**	**p**	**Crude OR (95% CI)**	**p**	**Adj. OR (95% CI)**	**p**
Region								
Southern	1		1		1			
Highlands	0.61 (0.23-1.60)	0.291	0.87 (0.28-2.69)	0.800	0.71 (0.22-2.33)	0.548		
Momase	4.77 (1.40-16.25)	0.016	4.81 (1.36-16.95)	0.018	1.21 (0.35-4.20)	0.754		
Islands	0.90 (0.35-2.31)	0.822	1.01 (0.37-2.72)	0.988	1.30 (0.49-3.45)	0.577		
0-1300	1.00				1			
1300-1699	0.34 (0.15-0.78)	0.014			0.52 (1.00-2.03)	0.322		
1700+	0.71 (0.17-3.02)	0.619			0.74 (0.52-4.54)	0.728		
Road	1.91 (0.84-4.34)	0.116			1.89 (0.72-4.94)	0.178		
Air only	0.19 (0.08-0.44)	0.001	0.26 (0.10-0.68)	0.009	0.19 (0.05-0.77)	0.023	0.19 (0.05-0.76)	0.021
Boat only	1.49 (0.77-2.89)	0.222			1.41 (0.66-3.00)	0.349		
2005	1				1			
2006	1.75 (0.72-4.21)	0.197			1.16 (0.52-2.57)	0.700		
2007	1.90 (0.52-2.83)	0.230			1.63 (0.67-3.95)	0.262		
1 person	1				1			
>1 person	4.70 (2.22-9.95)	0.001	5.35 (2.61-10.96)	<0.001	5.09 (2.35-11.03)	<0.001	5.26 (2.55-10.87)	<0.001
Rain	1				1			
End of rain	1.75 (0.72-4.21)	0.197			1.16 (0.52-2.57)	0.700		
Dry	1.90 (0.64-5.66)	0.230			1.63 (0.67-3.95)	0.262		

Univariate and multivariate models based on weighted country-wide post-distribution survey.

**Figure 3:**
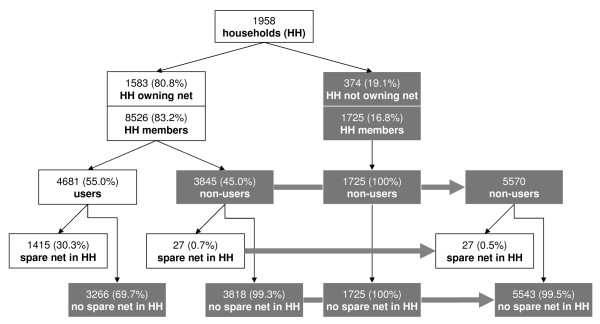
**Availability of nets to users and non-users of mosquito nets.** Data from country-wide post-distribution survey.

Net ownership was the principal determinant of net use. Out of 5570 surveyed household members who did not sleep under a mosquito net, 1725 (31.0%) lived in a household that did not own any net. The remaining 3845 (69.0%) non-users had a net in their household but only 27 (0.7% or 0.5% of all non-users) of them would have had access to an unused spare net (35 in Momase, 3 in the Islands) (Figure 3). Conversely, most unused nets were found in households in which everybody already slept under a net. In households with at least one net per household member, everybody slept under a net. A multivariate logistic regression model adjusting for the effect of age, number of household members, campaign year and village accessibility identified the number of people in a household (OR 0.79, 95% CI 0.67-0.92, p = 0.006) and older age (5–14 years OR 1.73, 95% CI 1.09-2.73, p = 0.022; 15+ years OR 1.53, 95% CI 1.06-2.21, p = 0.025) as being correlated with usage of a mosquito net in households owning a net. Due to collinearity, net ownership could not be included in the multivariate model. Season (rain, end of rain, dry) at the time of the interview, sex and pregnancy were no significant predictors of net use in the univariate analysis and therefore not included in the multivariate model. For LLINs in particular, ownership was the only significant determinant.

A total of 3,639 mosquito nets were found in sampled households, 2,320 (63.8%) of which had been used the previous night. Non-LLINs were used more often than LLINs (68.4% vs. 62.1%, p = 0.001). Household heads most frequently mentioned that a net was not being used because it was being spared for later use, either for a new house, a visitor, or a particular person who was absent at the time of the survey (32.8%). A total of 17.7% of the nets were not being used because they were considered “expired”, e.g. because it was damaged, had too many holes, was old or too dirty. Other frequently cited reasons included: perceived absence of mosquitoes (11.9%), feeling too hot under the net (11.0%) or simply a dislike or complacency about the use of mosquito nets (11.0%).

### Before-after assessment of ownership and usage

**Figure 4:**
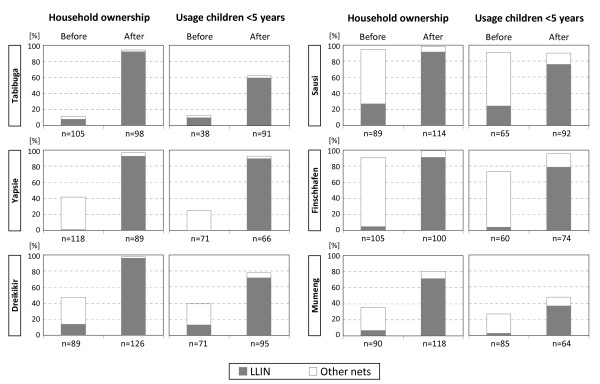
**Household ownership and usage of nets by children under five years before and after the distribution campaign in six sentinel sites.** Includes survey data from three to four villages per site. All difference are statistically significant at p < 0.001.

In the before-after survey in six sentinel sites, pooled household ownership of LLINs increased significantly from an average of 9.4% (7.2-12) prior to the distribution to 88.7% (86.0-91.0) one year later (p < 0.001). The overall increase in any type of net was less pronounced but still significant (p < 0.001), i.e. from 52.7% (48.6-56.8) to 94.1% (92.0-95.8). In five of the six sites, household LLIN ownership of over 90% was achieved. Two sites (Sausi and Finschhafen) were found to have high pre-distribution levels of ownership of nets (>90% in both sites), mostly non-LLINs. In these sites, the campaign led to only minimal changes in overall net ownership but still to a significant increase in ownership of LLINs (p < 0.001) (Figure 4).

In the target group of children under five years of age, usage of LLINs increased from an average of 8.2% (5.7-11.4) to 67.0% (62.6-71.2) and usage of any type of net from 44.6% (39.6.49.7) to 76.1% (72.1-79.9). Usage of LLINs by pregnant women increased from 9.4% (3.1-20.7) to 61.7% (46.4-75.5); usage of any net from 39.6% (26.5-54.0) to 72.3% (57.4-84.4). Overall, LLINs were used by 5.5% (4.7-6.4) of household members before and by 55.1% (53.4-56.8) after the campaign; nets of any type by 37.3% (35.5-39.1) before and 66.7% (65.1-68.3) thereafter. All changes were statistically significant (p ≤ 0.001). In general, usage increased alongside ownership in all sites (Figure 4). In Sausi and Finschhafen, high pre-distribution usage of any type of net but low LLIN usage was found. In these two locations, the distribution campaign resulted in a significant increase in the use of LLINs suggesting that most “old” nets were replaced by the newly distributed LLINs (Figure 4). Two sites in highland (Tabibuga) and highland fringe (Mumeng) areas showed the least improvement in usage after the distribution. In the case of Mumeng, this was coupled with comparably low post-campaign ownership (71.2% LLIN) and a higher person/net ratio than in the other sentinel sites (3.5, 95% CI 3.3-3.7 vs. average of 2.2, 95% CI 2.18-2.27 in other sites). In Tabibuga, household ownership was high after the campaign and the person/net ration was comparable to the other sites (2.6, 95% CI 2.5-2.8).

## Discussion

A single round of free LLIN distribution resulted in a significant increase in ownership and usage of mosquito nets in PNG. While the Global Fund supported distribution campaign failed to reach the 80% ownership and usage targets for LLINs, this should not divert attention from the fact that after the campaign, over 80% of households did own some type of mosquito net. While “only” 64.6% of households owned a LLIN, this nevertheless represents a significant increase from pre-distribution LLIN coverage (estimated at below 10%, *cf.* sentinel site data).

While the use of nets in general (51.8% in children under five years) and LLINs in particular (39.5% in children under five years) remained relatively low, this study proves that the distribution resulted in a major increase in net usage in locations where enough nets were available to household members. While it was not possible to collect country-wide pre-distribution data in the frame of this evaluation, data from sentinel sites show clearly the significant changes in both ownership (almost ten-fold increase for LLINs) and usage following the campaign. By comparison, in Kenya, where ITNs had been used for much longer, a similar (though targeted) campaign achieved merely 50.7% household ownership of ITNs and 51.7% usage by children under the age of five [24].

All evidence from this study suggests that under-supply of nets is the primary contributor to low usage, with under-supply determined chiefly by accessibility of households (villages) and household size. Most non-users found in this survey (99.5%) did not live in a household that would have had an extra net. Only 0.5% of the non-users could therefore (in the most optimistic scenario) have used one of the existing nets. Consequently, more nets (and sustainable supply channels) are required at least as urgently as the creation of awareness of the benefits of using nets. Awareness creation without extra campaign supply could help to increase net usage in areas with complementary sources of nets. However, currently nets are not commonly available in rural areas of PNG, particularly not at a subsidized price. Other factors not identified in this analysis may certainly also have contributed to non-usage, at least indirectly. For instance, some households reported as not owning a mosquito net may have received a net but for some reasons disposed of it. Reasons for non-use and non-ownership can be expected to vary greatly depending on the local setting. Efforts to increase net use should be based on detailed local evidence of why people don’t use mosquito nets. Yet thus far there is a dearth of dedicated and well-designed studies on reasons for not using a net as evidenced by a recently published review of the published literature [25]. Tentatively, this review found that discomfort due to heat and an absence of mosquitoes were frequently identified reasons for non-use [25], while in this survey such reasons were mentioned less frequently. Some people evidently treasured the mosquito net as a valuable household asset (sometimes belonging to a particular person) yet its intended use for protection against malaria-transmitting mosquitoes often appeared less important.

The insufficient (and very heterogeneous) supply of mosquito nets may be attributed to various factors related to the delivery system as a whole. Accessibility of villages was identified as major determinant of net ownership. This is unlikely to change over time and needs to be considered in the operational plans during subsequent distribution rounds. While the distribution ratio was set at 2.5 persons per LLIN, the nets procured could only achieve a ratio of 2.86/LLIN (6,641,268 population/2,321,000 nets). The procurements were based on population data from the year 2000 census plus an estimated population growth rate, which accounted for regional differences in growth but disregarded population movements within PNG. According to official figures, 2,005,052 LLINs were then distributed to 6,641,268 people resulting in an operational rate of 3.31/LLIN. However, this survey found that one LLIN was available per 4.1 persons only. Extrapolated, this would mean that either, the population to which the nets were distributed was 1.58 Mio (24%) higher than estimated. Or, that out of the 2 Mio nets officially distributed, 385,000 did not find their way to the target population. Or, that both factors played a role. In official records, 232,000 nets were recorded as not distributed and 84,000 nets were reported stolen (Anna Maalsen, NDoH, personal communication). Inaccuracy in distribution records and failure to supply the national level with final reports may also have contributed to the discrepancies between official reports and survey findings.

Remarkable differences between provinces, villages, and households were discovered. In general, they suggest no between-region difference in campaign penetration but significant differences in pre-distribution coverage and heterogeneity in the implementation of the distribution at provincial or village levels. In essence, while the distribution was carried out in every province, some villages still missed out and in villages covered by the campaign, some households still did not own a net. Reasons for this may be manifold and include a lack of sufficient numbers of nets for distribution as suggested by anecdotal evidence from malaria control officers. Conversely, a considerable number of households owned more nets than needed (Figure 3) which may be attributed to campaign teams either not considering pre-distribution net ownership or over-supplying of nets during the distribution. At higher altitudes, low ownership and usage may be a lesser problem for malaria control due to lower transmission during normal years. However, in highly endemic lowland areas such as the Islands provinces, low usage (29.3% overall net usage) may severely jeopardize malaria control efforts.

Considering the enormous operational challenges faced by health programmes (and any other outreach programmes) in PNG as a consequence of the currently largely dysfunctional health system as well as the difficult accessibility of large parts of the country, the 80% targets for 2009 may have been overly ambitious, even in comparison with global targets adopted by Roll Back Malaria [26]. Until the Global Fund Round 3 grant, the use of ITNs was never actively promoted through social marketing, subsidized or free distribution, or any other means on a national scale. While PNG National Health Plans [15,27] explicitly mentioned the use of mosquito nets, provision of nets was limited to local distributions by non-governmental organizations or targeted at pregnant mothers through health facilities. Pre-campaign net coverage was therefore mostly low and patchy [28,29]. The PNG experience hence raises questions about the appropriateness of applying global targets to local programmes without due consideration of the specific local circumstances (or in the absence of sufficient contextual evidence of the pre-implementation situation). This may be particularly problematic under performance-based funding arrangements such as Global Fund grants in which case it may jeopardize the continuation of a malaria control programme. Both, programme planners and funding agencies might in such situations preferably agree upon less ambitious targets in order to prevent a foreseeable failure of the programme.

This study is the first of its kind conducted in PNG, providing evidence of country-wide coverage with mosquito nets for malaria control. Due to the late commissioning of this evaluation (in year 4 of the Round 3 grant), it was not possible to collect country-wide pre-distribution data and selected sentinel sites are therefore used as proxy. The discrepancies between post-distribution data from the country-wide survey and from sentinel sites may in part be attributed to problems with the distribution particularly in the early phase of the Global Fund supported programme [18], and to a loss of nets over time. The country wide survey included areas that had received nets as long as four years prior to the survey while post-distribution surveys in sentinel sites were done within less than one year of the distribution. In addition, it cannot be ruled out that the research team’s focus on the sentinel sites may have encouraged a more thorough implementation of the programme in these locations and influenced the responses of interviewees. To reduce any reporting bias in self-reported net usage, independent household samples were drawn (to minimize the chance of asking the same question twice in the same household), the existence of nets was verified, and usage was recorded for each net individually. Interviewees were asked to identify the user(s) of each of the nets, which was then cross-checked with that person’s recorded presence the previous night.

## Conclusions

With one round of free distribution of LLINs, the PNG National Malaria Control Programme managed to significantly increase mosquito net ownership across the country. However, the implementation environment in PNG is challenging and programme targets were missed most probably as a result of operational constraints and flaws in the distribution process. An overall insufficient number of nets coupled with a heterogeneous distribution are the main reasons for overall low usage rates. In the frame of performance based funding schemes, the definition of programmatic targets needs to be considered very carefully. Overly ambitious targets may jeopardize large programmes, and inability to adapt targets to changing realities lays the foundation for programmes to fail, particularly in quickly changing, challenging environments. Funding bodies as well as implementing agencies need to take these realities into consideration when planning large-scale health programmes.

## Competing interests

The authors declare that they have no competing interests.

## Authors' contributions

MWH developed the study protocol and survey tools, supervised the data collection, analysed the data and wrote the manuscript. GG contributed to the development of survey tools, led the data collection, participated in data analysis and writing of the manuscript. NL developed the study database, organized data entry and contributed to data cleaning. LM revised the study protocol, supported the data collection and revised the manuscript. IM and PMS conceived the overall evaluation programme and supported data collection, analysis and manuscript writing. All authors (except GG) read and approved the final manuscript.

## Authors' information

This manuscript is dedicated to Mr. Gibson Gideon (GG), one of the principal contributors to this work, who during the course of conducting a follow-up malaria survey in West New Britain province disappeared without trace. Gibson and four other IMR colleagues who accompanied him are currently listed as ‘missing persons’.
